# Bilateral optic neuritis in a 26-year-old man with common variable immunodeficiency: a case report

**DOI:** 10.1186/1752-1947-5-319

**Published:** 2011-07-19

**Authors:** Angel P Sempere, ML Tahoces, Susana Palao-Duarte, Alfonso Garcia-Perez

**Affiliations:** 1 Neurology Department, Hospital General Universitario de Alicante, Alicante, Spain; 2 Hematology Service, Hospital Marina Baixa, Villajoyosa, Spain

## Abstract

**Introduction:**

Common variable immunodeficiency encompasses a group of heterogeneous conditions linked by a lack of immunoglobulin production and primary antibody failure. Although primary immunodeficiencies are typically characterized by recurrent infections, autoimmune manifestations have increasingly been recognized. Neurological complications are extremely rare and to the best of our knowledge optic neuritis has not been described previously. We report the case of a patient with common variable immunodeficiency who developed loss of vision secondary to bilateral optic neuritis.

**Case presentation:**

A 26-year-old Caucasian man with a diagnosis of common variable immunodeficiency presented to our facility with loss of vision secondary to bilateral optic neuritis. Results of a thorough study for infectious, neoplastic and autoimmune diseases were negative. Our patient was treated with intravenous methylprednisolone with almost complete improvement and he remained asymptomatic at a 12-month follow-up.

**Conclusions:**

Bilateral optic neuritis should be added to the list of autoimmune disorders related to common variable immunodeficiency. If a patient with common variable immunodeficiency experiences loss of vision, the possibility of bilateral optic neuritis should be considered as rapid initiation of high-dose corticosteroids may improve visual recovery.

## Introduction

Common variable immunodeficiency (CVID) encompasses a group of heterogeneous conditions linked by a lack of immunoglobulin production and primary antibody failure. Although primary immunodeficiencies are typically characterized by recurrent infections, autoimmune manifestations have increasingly been recognized [1]. The commonest autoimmune conditions in CVID are hematologic cytopenias. Neurological complications are extremely rare [2] and to the best of our knowledge optic neuritis has not been described previously. We report the case of a patient with CVID who developed loss of vision secondary to bilateral optic neuritis.

## Case presentation

A 26-year-old man with a diagnosis of CVID presented to our facility with loss of vision in both eyes. The first manifestation of CVID was hemolytic anemia at the age of 14, when the diagnosis was made. Later, he experienced several episodes of autoimmune thrombocytopenia and neutropenia. Since diagnosis, his only serious infectious complication had been an episode of salmonella sepsis at the age of 23. Actual treatment included intravenous immunoglobulin (0.4 g/kg every three weeks) and prednisone (10 mg daily).

On presentation, our patient reported progressive loss of vision that had begun five days earlier. He also had mild pain with eye movement. His visual acuity was 0.1 with reduced color perception, and both pupils showed a poor response to light stimulation. A confrontational visual field examination showed a diffusely decreased visual field in both eyes. Results of a slit-lamp examination were negative for uveitis. On funduscopic examination, his optic disk appeared normal. He was afebrile, and the results of general physical and neurological examinations were normal. There was no family history of eye disorders.

An MRI of the brain, orbit and spine revealed a mild enhancement of both optic nerves without other abnormalities. Pattern-shift visual evoked potentials showed marked delay of the P100 component from both eyes and attenuation of P100 wave amplitude. Laboratory investigation results revealed a normal blood count, and normal levels of liver enzymes, serum creatinine, angiotensin-converting enzyme, erythrocyte sedimentation rate, vitamin B12, folate and thyroid hormones. Serum levels of immunoglobulin were 1590 mg/100 mL, < 6 mg/100 mL, and 380 mg/100 mL for IgG, IgA and IgM, respectively. Serum serologies for Lyme, syphilis, *Brucella*, viral hepatitis, *Bartonella henselae *and HIV were negative. Test results for anti-nuclear antibodies, rheumatoid factor, anti-nuclear cytoplasmic antibodies, anti-phospholipid antibodies, anti-CV2, and anti-neuromyelitis optica (NMO) antibodies were also all negative. Fluorescein angiography showed no signs of retinal vasculitis. In addition, a chest and abdominal computed tomography (CT) scan and a whole-body positron emission tomography (PET) scan were unrevealing. A genetic study for Leber's hereditary optic neuropathy was also negative.

Cerebral spinal fluid (CSF) analysis revealed normal protein and glucose levels and a white cell count of 2 cells/mm^3 ^for all lymphocytes. No oligoclonal bands were present. No malignant cells were observed, and a microbiological investigation including CSF bacterial, fungal and mycobacterial cultures was negative. In addition, CSF PCR tests for enteroviruses, Herpes simplex virus, varicella zoster, Epstein-Barr, and cytomegalovirus were all negative.

Our patient was diagnosed with bilateral optic neuritis and treated with intravenous methylprednisolone (1000 mg/day for five days) and a tapering course of oral prednisone. Two weeks later, his visual acuity was one in the right eye and 0.6 in the left eye. Our patient remained asymptomatic at 12-month follow-up, when visual acuity was one in the right eye and 0.8 in the left eye.

## Discussion

Bilateral simultaneous optic neuritis is uncommon in adults [3]. In our patient, there was no evidence of a toxic, infectious or neoplastic disorder. Sarcoidosis seemed unlikely in view of the negative chest CT and PET scan results as well as the normal serum angiotensin-converting enzyme levels. Neuromyelitis optica also seemed improbable considering the normal spinal cord MRI, negative NMO antibodies and absence of recurrent episodes during the follow-up period [4]. Although CVID has been linked to uveitis [5] and retinal vasculitis [6], it has to date not been linked to optic neuritis.

Patients with CVID are predisposed to several autoimmune disorders, and bilateral optic neuritis should be added to the list. If a patient with CVID experiences loss of vision, the possibility of bilateral optic neuritis should be considered because rapid initiation of high-dose corticosteroids may improve visual recovery.

## Conclusions

This case report describes an unreported complication of common variable immunodeficiency: bilateral optic neuritis, linked to the autoimmune manifestation of the disease. If a patient with common variable immunodeficiency experiences loss of vision, the possibility of bilateral optic neuritis should be considered.

## Consent

Written informed consent was obtained from the patient for publication of this case report and accompanying images. A copy of the written consent is available for review by the Editor-in-Chief of this journal.

## Competing interests

The authors declare that they have no competing interests.

## Authors' contributions

APS and MLT conceived the study and drafted the manuscript, SPD and AGP helped to draft the manuscript. All authors have read and approved the final draft.
